# Acute Responses to Forearm Compression of Blood Lactate Accumulation, Heart Rate, Perceived Exertion, and Muscle Pain in Elite Climbers

**DOI:** 10.3389/fphys.2018.00605

**Published:** 2018-05-23

**Authors:** Florian Azad Engel, Billy Sperlich, Urs Stöcker, Peter Wolf, Volker Schöffl, Lars Donath

**Affiliations:** ^1^Department Movement and Training Science, Institute of Sport and Sport Science, Universität Heidelberg, Heidelberg, Germany; ^2^Integrative and Experimental Training Science, Department of Sport Science, Universität Würzburg, Würzburg, Germany; ^3^Ressort Leistungssport, Deutscher Alpenverein e.V., Munich, Germany; ^4^Sensory-Motor Systems Lab, Department of Health Sciences and Technology, ETH Zürich, Zurich, Switzerland; ^5^Department of Sports Orthopedics and Sports Medicine, Klinikum Bamberg, Bamberg, Germany; ^6^Department of Intervention Research in Exercise Training, German Sport University Cologne, Cologne, Germany

**Keywords:** acute response, climbing, compression garments, clothing, external pressure

## Abstract

**Objectives:** To evaluate the immediate responses to forearm compression of blood lactate concentration, heart rate, perceived exertion and local forearm muscle pain during severe climbing in elite climbers.

**Method:** Seven elite climbers (18 ± 2 years; 164 ± 5 cm; 57.8 ± 5.3 kg) performed 3 × 3 climbing bouts with maximal intensity on a distinct 8 m boulder wall (lead grade: 7a–8b) in a single blinded, placebo-controlled cross-over design, wearing either forearm sleeves with compression (verum-compression) or placebo forearm sleeves with no compression (falsum-compression). Each climber’s heart rate was recorded during and capillary blood lactate concentration, perceived exertion and forearm muscle pain were assessed directly after climbing.

**Result:** Heart rate (*p* = 0.45, $\eta_{p}^{2}$ = 0.12), blood lactate concentrations (*p* = 0.44, $\eta_{p}^{2}$ = 0.10), perceived exertion levels (*p* = 0.51, $\eta_{p}^{2}$ = 0.08) and pain perception (*p* = 0.67, $\eta_{p}^{2}$ = 0.03) were not affected by forearm compression. No condition × time interaction effect (compression × time) occurred for heart rate (*p* = 0.66, $\eta_{p}^{2}$ = 0.04), blood lactate concentration (*p* = 0.70, $\eta_{p}^{2}$ = 0.02), perceived exertion (*p* = 0.20, $\eta_{p}^{2}$ = 0.26) and pain perception (*p* = 0.62, $\eta_{p}^{2}$ = 0.04).

**Conclusion:** In elite climbers performing severe climbing bouts, sleeves with forearm compression do not alter blood lactate concentration, heart rate, perceived exertion and local forearm muscle pain.

## Introduction

Within the last decades, climbing and bouldering have gained increasing popularity (Schweizer, 2012). Typical ascents in sport climbing last between 2 and 7 min and climbers utilize approximately 20–25 ml/kg/min of oxygen (Watts, 2004). Climbing is explicitly challenging for upper limbs since handgrip endurance and strength decrease while climbing severely, due to muscle contraction-induced ischemia in forearm flexor muscles (Watts, 2004; Fryer et al., 2016). Thus, ischemia associated with a decline in muscle oxygenation causes muscle fatigue in forearm extensor muscles (Murthy et al., 2001). Consequently, climbers seek methods to enhance performance and recovery. A simple, socially, ethically acceptable, and inexpensive ergogenic strategy in this context would be of considerable value to climbers.

In previous investigations arterial perfusion of the human forearm at rest increases more than twofold while exposed to external compression (13–23 mmHg) (Bochmann et al., 2005). Since the protocol of Bochmann et al. (2005) was performed (a) at rest and with the arm at heart level and (b) not during intense climbing involving arm movements above the heart level, it is not clear whether the application of external compression during climbing increases arterial perfusion. Based on studies investigating lower limb compression, a climber might benefit from improved hemodynamics, including improved oxygenation, with lower osmotic pressure, altogether reducing ischemia (Kraemer et al., 2001) with an increase in venous blood return (Lawrence and Kakkar, 1980; Gandhi et al., 1984). An external mechanical pressure causes redistribution of blood volume from the superficial to the deep venous system (Kraemer et al., 2001), thereby reducing swelling of damaged muscle tissue by facilitating lymphatic drainage, and reducing osmotic pressure (Kraemer et al., 2001). In contrast, previous research demonstrated that compression applied to lower limbs did not necessarily improve lower limb blood flow at rest (Lawrence and Kakkar, 1980) or during intense exercise (Born et al., 2014).

Additionally, recent reviews attributed improved recovery through compression clothing to augmented clearance of blood lactate and improved ratings of perceived exertions (Hill et al., 2014; Beliard et al., 2015; Engel et al., 2016). Furthermore, compression garments could provide a mechanical support to the limbs during exercise, which may reduce force decrements (Kraemer et al., 2001) which in turn, would be preferred by climbers, since handgrip strength and handgrip endurance decreases during competitive climbing (Watts, 2004).

Since there is evidence that arm compression may improve oxygenation and reduce ischemia (Bochmann et al., 2005), we aimed to analyze the impact of forearm compression sleeves on tissue oxygenation and muscle perfusion in the forearm muscles during climbing, using a portable near-infrared spectroscope (NIRS). Furthermore, the purpose of the study was to examine the acute responses to sleeves which exert forearm compression, on important variables related to climbing including blood lactate concentrations, heart rate, perceived exertion and forearm muscle pain, during and following intense climbing by elite climbers. However, implementation of the NIRS during climbing was not possible due to the risk of injury when falling, an unnatural grip feeling, and an overall lack of acceptance among the elite climbers.

## Materials and Methods

### Participants

Seven elite climbers (five female and two male) of the Swiss national team (age: 18 ± 2 years; height: 165 ± 5 cm; body-mass: 58 ± 5 kg; body mass index: 22 ± 2 kg/m^2^) voluntarily participated in the study. All climbers were accustomed to five climbing sessions per week lasting 1–3 h, and had been frequently involved in climbing competitions at an international level for at least 2 years (Training time per week: 16.1 ± 5.6 h; training experience: 8.7 ± 2.2 years; Ape-index (Watts et al., 2003): 3.9 ± 3.2 cm, climbing level lead: 8a – 8c+ on “French scale”). Each participant, or their legal guardian, signed a written consent form before participating. The study protocol complied with the code of ethics for human experimentation published by the World Medical Association in the Declaration of Helsinki (World Medical Association [WMA], 2013), and was pre-approved by the institutional ethics committee of the Department of Sport, Exercise and Health, University of Basel, Switzerland. All participants completed a health screening questionnaire, and individuals with a history of musculoskeletal injury and inflammatory disorders were excluded from participation. All climbers were asked to rest, and to refrain from strenuous exercise in the 48 h preceding each session, and for 48 h following the climbing protocol. In addition, participants were required to refrain from using any recovery strategy (e.g., massage) for the duration of the investigation.

### Experimental Overview

The present study was conducted as a single blinded, placebo-controlled cross-over study. Both verum-compression and falsum-compression forearm sleeves were randomly and single-blindly assigned to the participants. The experimental climbing sessions were conducted within a period of 14 days, at least 48 h apart in order to guarantee adequate recovery. Participants completed a climbing session with verum-compression and a climbing session with falsum-compression forearm sleeves in a randomized order. Each session comprised 3 × 3 climbing bouts with maximal intensity on a distinct 8 m boulder wall (lead grade: 7a–8b). At rest, and 60 s following each climbing bout, capillary blood lactate, heart rate, perceived exertion on the Borg 6–20 scale (RPE) (Borg, 1982), and forearm muscle pain on the visual analog scale (VAS) (Huskisson, 1982) were recorded.

### Climbing Protocol

The climbing took place in an indoor climbing center in Switzerland. The climbing protocol commenced with a 30-min standardized warm-up, including a 2 × 20 m horizontal traversing of a slightly overhanging climbing wall (60–80 easy grips); 3 × 4 pull-ups, 4 burpees, 4 dips, 2 × vertical traversing of ∼8 m climbing wall Fontainebleau 6a, 2 × vertical traversing of ∼8 m climbing wall Fontainebleau 6b, and 2 × vertical traversing of ∼8 m climbing wall Fontainebleau 6c–7b. Following a warm-up the climbers performed the actual climbs, including 3 × 3 boulders, as rapidly as possible, each on a 45° overhanging and an 8 m-high boulder wall (lead grade: 7a–8b), comprising approximately 40 moves per bout. The 3 × 3 climbing bouts were performed adhering to current world cup rules (International Federation of Sport Climbing, 2017), which provide a 4 min time limit for each boulder. Climbers were instructed to climb as fast as possible; if the 4 min were exceeded, climbers had to terminate the climb, and climb or jump down. Durations of climbs were not documented. The climbers rested for 4 min after each boulder and 6 min after each series. In the case of a fall, the climbers had to directly pursue bouldering in order to complete the number of moves within the allotted 4 min. Each boulder route was individually pre-defined by the Swiss national coach to meet the individual performance level of each athlete. Verbal encouragement to each climber was provided by the coach.

### Variables

For analyses of capillary blood lactate concentrations, 20 μL of capillary blood was collected from the right earlobe. The pretest blood sample was drawn after the warm-up and 120 s before the start of the first boulder. The post climbing sample was drawn 60 s following the last climb of each series (60 s after each climber reached the final hold with both hands). Procedures were consistent among all participants. For the final analysis, the mean of the three post-test values was analyzed. Lactate concentration was analyzed through an enzymatic-amperometric procedure using the Super GL ambulance (Dr. Müller Gerätebau^®^, Freital, Germany). Under our laboratory conditions, the coefficient of variation for repeated measurements of lactate concentration is routinely 1.6% at a concentration of 12 mmol/l.

Each climber’s heart rate was recorded continuously at 0.5 s intervals during the warm-up, as well as during and following climbing via an electrode chest belt and an RS800CX watch (Polar electro^®^, Kempele, Finland). For the final analysis, a mean heart rate of each 60 s interval was analyzed. The pretest value consisted of a 60 s interval following the warm-up, and 120 s before the start of the first climb. The posttest value represents the mean of nine 60 s intervals following each climb of the 3 × 3 climbing bouts (60 s after climbers had reached the final hold with both hands).

Immediately after each climb, all climbers rated their perceived exertion during climbing on the Borg 6–20 scale (Borg, 1982). The pretest value was measured following the warm-up and refers to perceived exertion in the warm-up. The posttest value represents the mean of the nine ratings following each of the 3 × 3 climbing bouts.

A VAS was applied in order to evaluate perceived pain levels (Huskisson, 1982). A 10-cm VAS was presented to each athlete in order to rate their local muscle pain in the forearm. The pretest value was documented subsequent to the warm-up, and refers to the local muscle pain experienced from the warm-up. A posttest measurement was taken immediately following the last of the 3 × 3 climbing bouts, representing the local muscle pain experienced from climbing bouts.

### Compression Sleeves

During warm-up and throughout the entire climbing protocols all climbers wore either verum-compression or falsum-compression forearm sleeves (both from VERTICS^®^, Wiesbaden, Germany). Both types of sleeve had the same look and could not be distinguished by the participants. The evaluator was aware about the level of compression. The participants were only informed that a novel product for climbing was being tested. The verum-compression garment comprised of 75% polyamide and 25% spandex. The falsum-compression forearm sleeves were custom made VERTICS^®^ Sleeves without spandex and did not have the capacity for compression. Both verum and falsum-compression sleeves covered the entire forearm, from the processus styloideus radii to the olecranon process. The verum-compression (VERTICS^®^ Sleeves) was fitted according to the manufacturer’s guidelines based upon the greatest forearm circumference, which was measured three times, with the mean being considered as the forearm circumference. The verum-compression sleeves exerted a graduated compression from distal to proximal. According to the manufacturer, the pressure of the verum-compression sleeves was 22.4 mmHg at the distal, decreasing to 12.4 mmHg at the proximal end.

### Statistical Analysis

All data are presented as mean ± standard deviation (SD). Normal distribution (Kolmogorov-Smirnov Test) and homogeneity of variance (Levene Test) were tested prior to further statistical analysis with no transformation necessary. Separate repeated measures analyses of variance (rANOVA) were computed for each variable (blood lactate, heart rate, perceived exertion and muscle pain) during verum and falsum conditions (condition effect) at pre and post testing. Main- and interaction-effects were considered significant with *p* < 0.05. To account for significant condition × time effects, Tukey *post hoc* tests were calculated for pairwise comparison. To estimate practical relevance, effect sizes (partial eta squared, $\eta_{p}^{2}$) calculated with $\eta_{p}^{2}$ ≥ 0.01 indicated small, ≥0.06 medium and ≥0.14 large effects. All analyses were performed using SPSS for Windows, version 21, (IBM, Armonk, United States).

## Results

### Blood Lactate Concentration and Heart Rate

**FIGURE 1:**
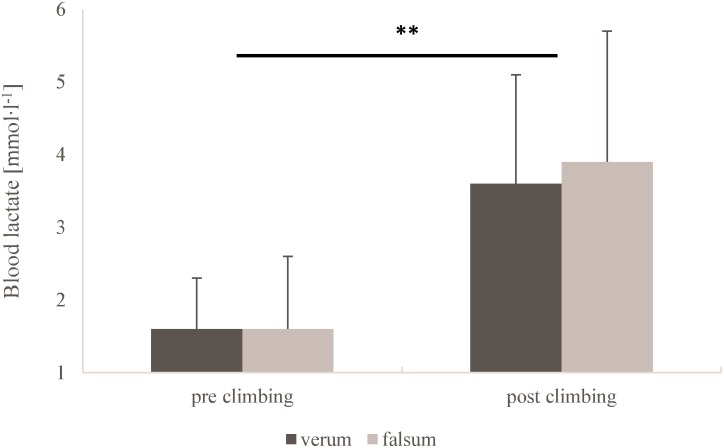
Capillary blood lactate concentrations for verum-compression (black) and falsum-compression (gray) condition at pre-climbing and post-climbing. **^∗∗^**Significant (*p* < 0.001, $\eta_{p}^{2}$ = 0.77) time effect as a response to climbing.

**FIGURE 2:**
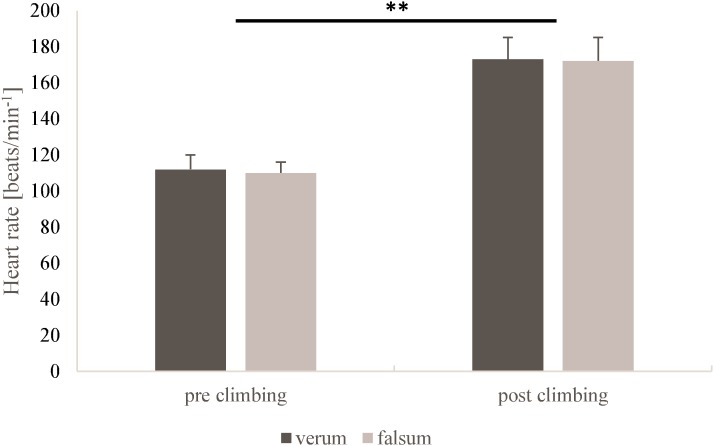
Heart rates for verum-compression (black) and falsum-compression (gray) condition at pre-climbing and post-climbing. **^∗∗^**Significant (*p* < 0.001, $\eta_{p}^{2}$ = 0.97) time effect as a response to climbing.

Blood lactate concentrations (*p* = 0.44, $\eta_{p}^{2}$ = 0.10) and heart rate (*p* = 0.45, $\eta_{p}^{2}$ = 0.12) did not differ between verum-compression and falsum-compression conditions. No interaction effects (condition × time) were found for blood lactate concentration (*p* = 0.70, $\eta_{p}^{2}$ = 0.02) nor for heart rate (*p* = 0.66, $\eta_{p}^{2}$ = 0.04). A large and significant time effect was observed for blood lactate concentration (*p* < 0.001, $\eta_{p}^{2}$ = 0.77) and heart rate (*p* < 0.001, $\eta_{p}^{2}$ = 0.97) as a response to climbing. (**Figures 1, 2**).

### Perceived Exertion and Muscle Pain

**FIGURE 3:**
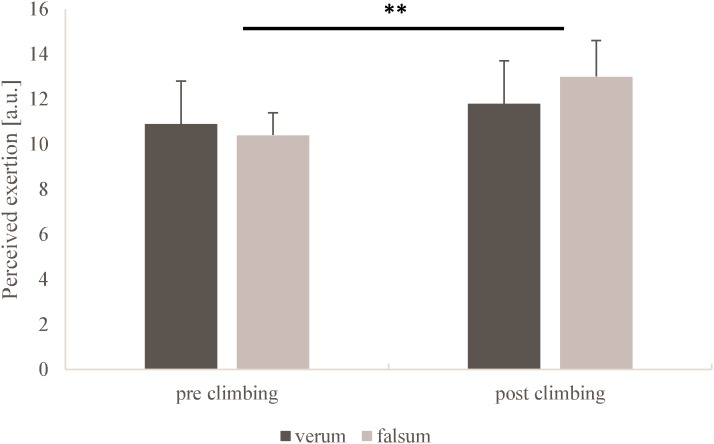
Rates of perceived exertion obtained from the Borg scale for verum-compression (black) and falsum-compression (gray) condition at pre-climbing and post-climbing. a.u., arbitrary units. **^∗∗^**Significant (*p* < 0.001, $\eta_{p}^{2}$ = 0.74) time effect as a response to climbing.

**FIGURE 4:**
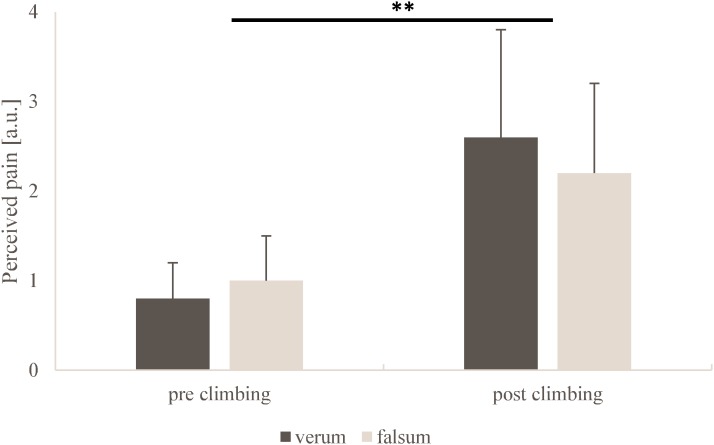
Pain perception obtained from the visual analog scale for verum-compression (black) and falsum-compression (gray) condition at pre-climbing and post-climbing. a.u. = arbitrary units. **^∗∗^**Significant (*p* = 0.01, $\eta_{p}^{2}$ = 0.66) time effect as a response to climbing.

RPE (*p* = 0.51, $\eta_{p}^{2}$ = 0.08) and pain perception (*p* = 0.67, $\eta_{p}^{2}$ = 0.03) did not differ between verum-compression and falsum-compression. A very large but non-significant interaction (condition × time) was observed for RPE (*p* = 0.20, $\eta_{p}^{2}$ = 0.26). No interaction was observed for pain perception (*p* = 0.62, $\eta_{p}^{2}$ = 0.04). A large and significant time effect was found for RPE (*p* < 0.001, $\eta_{p}^{2}$ = 0.74) and pain perception (*p* = 0.01, $\eta_{p}^{2}$ = 0.66) as a response to climbing. (**Figures 3, 4**).

## Discussion

The present randomized-placebo-controlled and single blinded cross-over study aimed to investigate the acute responses of forearm compression sleeves on heart rate response, blood lactate concentration, perceived exertion and muscle pain during severe climbing in elite climbers.

Altogether, the present investigation revealed no influence of graduated forearm compression sleeves on blood lactate concentration, heart rate, perceived exertion and local muscle pain following climbing. Large and significant time effects were observed for all measured variables (blood lactate concentration, heart rate, perceived exertion, and muscle pain) as a response to climbing, thus demonstrating the high intensity effort during the climbing bouts.

In contrast to the present data, a previous study demonstrated that a long-sleeve compression shirt worn during 3 × 3 min of double poling sprints lowered post exercise blood lactate concentrations (Sperlich et al., 2014). In this context compression applied to leg muscles may diminish blood lactate concentrations following high-intensity endurance exercise, thus retaining lactate in the muscle (Berry and McMurray, 1987; Chatard et al., 2004). In the present study, the compression sleeves covered only the forearms, representing a relatively small percentage of the body surface. The small area of exposure might explain why we did not find any differences in blood lactate concentrations between falsum and verum-compression. However, in line with our study, upper body compression worn during bench presses (50% of one repetition maximum), also did not alter blood lactate concentration and performance variables (Martorelli et al., 2015) when compared to non-compression exercise.

Nevertheless, neuromuscular and circulatory demands during climbing rely mainly on forearm muscles (Schweizer, 2012). Thus, extensive climbing or high-intensity competitions lead to ischemia-induced muscle fatigue in the forearm muscles (Schweizer, 2012). More rapid between-trial and between-day recovery, as well as countermeasures of muscle fatigue during climbing, are paramount for successful climbing (Schweizer, 2012). Despite the very small but homogeneous sample of the Swiss elite climbers, we only found acute responses of the verum forearm compression regarding blood lactate accumulation and the perceived exertion level. Athletes who wore verum-compression showed slightly lower lactate accumulation and less perceived exertion, but alterations were not significant, and effect sizes were moderate. Furthermore, the reduction of blood lactate levels and perceived exertion were considerably low, suggesting little practical relevance for climbers wearing forearm compression during competition. However, the latter finding seems to be particularly interesting, as perceived efforts or self-reported measures during training may predict an athlete’s training response (Saw et al., 2016) when compared to common objective variables. As a consequence, changes in physiologically and psychologically relevant training load indicators, such as blood lactate, heart rate, perceived exertion and pain perception could explain training responses in competitive sports.

The idea of “garment feel,” earlier examined by Kraemer et al. (1998), might have an ergogenic property during climbing. In the latter study, athletes were asked how they felt the compression garment affected their jumping performance without receiving feedback during repetitive jumps. Athletes reported a significant “perception of improvement” in favor of the compression garments. Corroboratively, vitality scores, arm function during daily life and muscle soreness (with range of motion and palpation) were rated more favorable (at 72–120 h post exercise) when wearing a compression sleeve, than without (MacRae et al., 2011). The improved feeling was attributed to elevated skin temperature and improved proprioception caused by compression (Kraemer et al., 1996; Chatard et al., 2004). Both elevated skin temperature and proprioception could in fact improve climbing, especially in a cold environment or on a cold surface.

### Limitations

A greater number of subjects would have given more statistical power to the data interpretation with less risk of calculating type 2 errors, however, the Swiss national team only consists of 14 athletes and the experimental sessions were conducted toward the end of season when many athletes had already finished their competition period. We also aimed to analyze a homogeneous sample of elite climbers, and a small sample size allowed us to increase availability, motivation and compliance of elite climbers in participating. Furthermore, we consider the application of potentially performance enhancing compression sleeves more relevant for elite than for lower level climbers. Due to the fact that climbing will be an Olympic discipline in 2020, the enhancement of athletic performance and recovery in climbing will become, and respectively is, increasingly important. In addition, the results, especially for blood lactate and muscle pain, were nearly identical in all subjects.

Although verum-compression and falsum-compression forearm sleeves looked the same, we cannot exclude the possibility that climbers could distinguish between the two sleeves. The participants were only informed that a novel product for climbing was being tested. The investigators did not articulate any suggestions at any time that one compression sleeve might be more effective than the other, in order to minimize any influence on the participants’ perceptions and intuitions concerning expected results.

The present study was conducted over a period of two weeks, and based on our data, we cannot judge the long-term effects of wearing compression sleeves.

## Conclusion

The present results revealed that forearm compression sleeves worn by elite climbers during severe climbing did not alter blood lactate concentrations, heart rates, rates of perceived exertion, or local muscle pain in forearms during and after a set of severe climbing.

## Author Contributions

LD, US, PW, VS, FE, and BS conceived and designed the experiments. LD, US, and PW performed the experiments. FE, BS, LD, and PW analyzed the data. FE, BS, and LD prepared the manuscript. All authors read and approved the final manuscript.

## Conflict of Interest Statement

The authors declare that the research was conducted in the absence of any commercial or financial relationships that could be construed as a potential conflict of interest.VERTICS^®^ had no role in the study design, data collection and analysis, decision to publish, or preparation of the manuscript.
